# A Review of Polarization-Sensitive Materials for Polarization Holography

**DOI:** 10.3390/ma13235562

**Published:** 2020-12-06

**Authors:** Yueyang Zhai, Li Cao, Ying Liu, Xiaodi Tan

**Affiliations:** 1 Innovation Research Institute of Frontier Science and Technology, Beihang University, Beijing 100191, China; yueyangzhai@buaa.edu.cn (Y.Z.); caoli722@buaa.edu.cn (L.C.); 2 School of Instrumentation and Optoelectronic Engineering, Beihang University, Beijing 100191, China; 3 Zhejiang Lab, Building 10, China Artificial Intelligence Town, 1818 Wenyi West Road, Hangzhou 310000, China; 4 Fujian Provincial Key Laboratory of Photonics Technology, College of Photonic and Electronic Engineering, Fujian Normal University, Fuzhou 350007, China

**Keywords:** polarization-sensitive material, holographic recording, photopolymer, photoinduced birefringence, azopolymer materials

## Abstract

Polarization holography has the unique capacity to record and retrieve the amplitude, phase, and polarization of light simultaneously in a polarization-sensitive recording material and has attracted widespread attention. Polarization holography is a noteworthy technology with potential applications in the fields of high-capacity data storage, polarization-controlled optical elements, and other related fields. The choice of its high-performance materials is particularly important. To further develop polarization holography applications and improve the quality of the information recorded (i.e., material sensitivity and resolution), a deeper understanding of such materials is needed. We present an overview of the polarization-sensitive materials, which introduced polarization holographic technology and the development of polarization holographic materials. The three main types of polarization holographic materials are described, including azopolymer materials, photopolymer material, and photorefractive polymer material. We examine the key contributions of each work and present many of the suggestions that have been made to improve the different polarization-sensitive photopolymer materials.

## 1. Introduction

Improvements in the capacity and reliability of data storage systems are urgently needed. One potential optical information storage technology uses a holographic approach in which recorded data are distributed throughout the volume of a thick medium with high density. Optical holographic recording technology can record information in a three-dimensional space versus traditional two-dimensional storage methods and offers a small volume, large capacity, and high density. In 1994, Heanue et al. first used holographic recording technology to realize the storage of digital images and compressed video data [1]. This high-density and large-capacity storage technology has attracted widespread attention.

**Figure 1:**
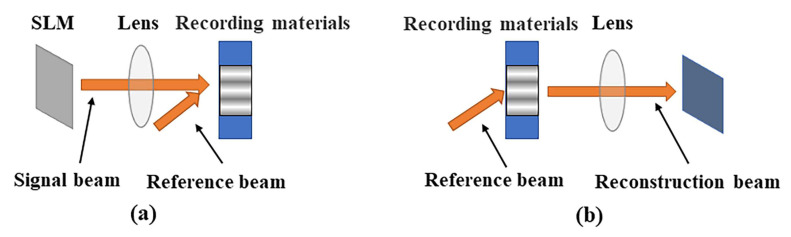
Principle of the holographic recording and reading process: (**a**) holographic recording process; (**b**) holographic reconstruction process, SLM, spatial light modulation.

In holographic data recording, the entire information contained in the light is stored at once as an optical interference pattern within a photosensitive optical material. Holographic storage technology usually uses the interference of light to record the interference fringes and uses the diffraction of light to reproduce the information of the recorded light. Holographic storage recording is achieved through the interference of two coherent laser beams irradiated at the same position in the photosensitive material. Figure 1 shows that the light emitted by the laser is divided into two beams: One beam carries data information through the spatial light modulator as information light, and the other beam is used as the reference light. When the information needs to be read, the hologram is irradiated with the same beam as the reference light to cause diffraction. The information can be reconstructed, and the initial image of the information can be read through the diffracted light [2]. As a recording medium, photosensitive materials greatly affect the holographic recording ability. Traditional holographic storage technology, however, considers only the phase and intensity information of light and ignores the polarization information. Polarization holography can employ waves with two different polarizations to record polarization states on the polarization-sensitive materials.

We describe a polarization holographic technology and reviewed three typical polarization-sensitive materials to more fully understand what is needed to optimize the performance for polarization holographic applications. More specifically, we describe the evolution of our understanding of what takes place inside these polarization materials and what happens during and after holographic recording. Finally, we propose a prospect for polarization-sensitive materials.

## 2. Polarization Holography

**Figure 2:**
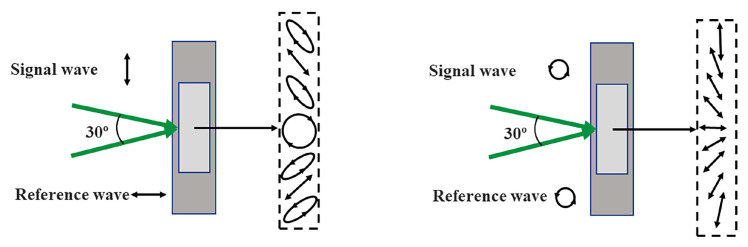
Interference field with the periodic change of the polarization states caused by two orthogonally linear polarized waves or two orthogonally circularly polarized waves.

The polarization properties of light and its anisotropy process in propagation increase as the degree of freedom, which can be controlled; this is called the polarization state. In 1965, Lohmann first proposed a method to record the polarization state of light with two orthogonal polarized beams [3]. In 1968, Foumey et al. described an experimental method to record and reproduce the polarization state of light, which verified Lohmann’s theory [4]. Holography that can record the polarization information of light is called polarization holography. Polarized holography is different from traditional holography, which can modulate the recording and reproduction of information using the polarization state. As presented in Figure 2, two orthogonally polarized waves interfere, forming an interference field with the periodic change of the polarization states.

**Figure 3:**
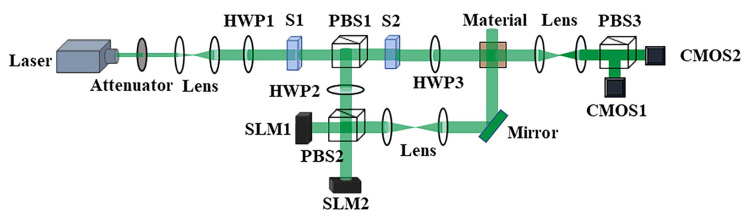
Polarization multichannel multiplexing device: S, HWP, PBS, SLM, and COMS are the shutter, half-wave plate, polarization beam splitter, spatial light modulation, and complementary metal oxide semiconductor sensor, respectively.

Since the concept of polarization holography was proposed, in 1974, Kakichashvili first gave a theoretical proof using the photoinduced anisotropy of polarization-sensitive materials for polarization holographic recording [5]. This was the first time the holographic method that uses the Weigert effect (photoinduced birefringence) to record the polarization state of the light field in a photosensitive material was presented. The interaction of the polarized light field with the photo-anisotropic material records the polarization state of the light field. Ordinary holographic recording materials cannot meet the requirements of polarized holography. This discovery caught the attention of researchers from polarization holographic recording to choose appropriate polarization-sensitive material. In 2009, Nikolova et al. adopted the Jones matrix to describe the recording and reproduction process of polarization holography, but the theory was established under the condition that two polarization interference lights were approximately parallel, which limited the development of this theory [6]. Because of recording condition restrictions, polarization holographic recording has not been widely studied and applied. In 2011, Kuroda et al. proposed a tensor-based polarization holographic theoretical model, which is applicable when two polarization interference lights are at any angle, which has wide universality [7]. Polarization holography technology is used mostly in polarization multichannel multiplexing, vector beam storage, polarization modulation and encoding, and optical component production. It has broad application prospects. For holographic data storage, polarization multichannel multiplexing will increase the one-dimensional polarization variable, as depicted in Figure 3. This means that holographic three-dimensional storage can be upgraded to four-dimensional systems, which effectively improves the storage density. With the development of polarization holography, polarization holography materials have also been continuously developed and optimized. To realize the real practical application of polarization holography theory, excellent polarization-sensitive materials are indispensable.

## 3. Polarization-Sensitive Material

In the development of polarization holographic storage technology, the most vital thing is to find suitable polarization-sensitive materials. Polarization-sensitive materials include organic materials and inorganic materials. Inorganic materials, such as photorefractive crystals, have a high diffraction efficiency and a short response time. These materials have harsh production conditions and high costs, however, which limits their marketability. They are not suitable for permanent storage of information because they can be erased [8,9]. Recently, metasurfaces also showed their superior capability in controlling the phase, amplitude, and polarization states of light [10,11]. Metasurfaces, which consist of an array of function-driven artificial meta-atoms, can be designed to exhibit highly anisotropic responses such that their interaction with electromagnetic fields is strongly polarization sensitive [12,13,14,15]. Although they have not been used in the study of polarization holography, some other materials have been found to be sensitive to polarization. Some special nanostructures with large field enhancements exhibit obvious polarization dependence, such as nanoscale gaps and nano-gold dimers [16,17]. The structural chirality of molecules gives rise to optical chirality. Ali Rafiei Miandashti et al. experimentally and theoretically observed photothermal chirality in gold nanoparticle helicoids [18]. In colloidally prepared gold helicoids, a polarization dependence was found in circular differential absorption and the maximum temperature of a small cluster of helical nanoparticles. There are many types of organic materials, and more attention has been given to photorefractive polymer materials, azopolymer materials, photochromic materials, and photopolymer materials.

An excellent volume holographic recording material needs to have the following characteristics: (i) The material should have sufficient optical quality to ensure that the loss of the beam during reading and writing is extremely small, and the surface of the material should have optical flatness to avoid distortion during the imaging process. The scattering rate of the material is extremely low, and for holographic storage, it needs to have sufficient thickness to achieve a high storage density. (ii) The recording wavelength of the material should match the wavelength of the laser, and it should be sensitive to light waves in the wavelength range. (iii) The material should be processed without traditional holographic processing, such as heat treatment or solvent processing, to reduce costs. (iv) For long-term recording, the material must be nonvolatile, that is subsequent holographic recording and reading will not destroy the recorded hologram. (v) The material must be able to maintain long-term stability within a certain temperature and humidity range to achieve sufficient storage life. In this part, we will describe three typical polarization holographic materials, which are azopolymer materials, photopolymer materials, and photorefractive polymer materials, in detail.

### 3.1. Azopolymer Materials

Azopolymers have good optical stability, good solubility, a low price, the ease of combination with polymers, high absorption strength, no fluorescence, and low phosphorescence loss. More importantly, their maximum absorption peak can be moved to the short-wave region (such as green and blue) through structural modification, which is expected to be a good polarization holographic recording material. The recording and retrieval capacity of polarization information in azopolymers is based on the birefringence induced by the preferential orientation of the chromophores in the material due to trans-cis-trans photoisomerization [19].

**Figure 4:**
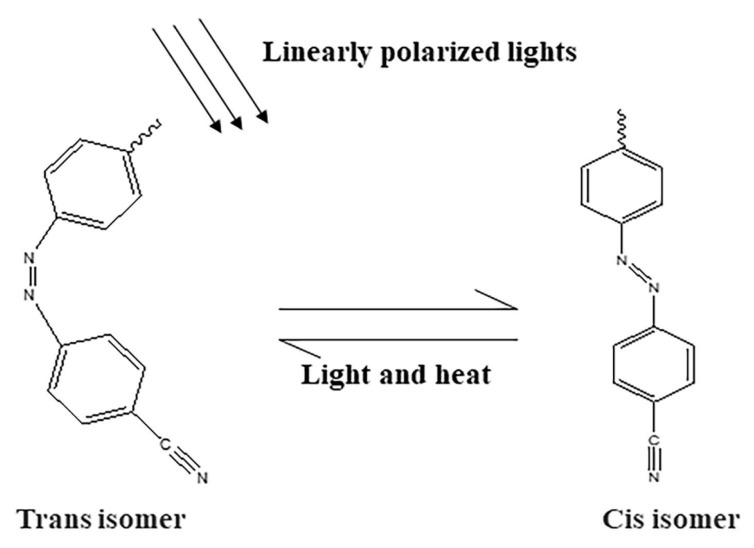
The cis-trans isomerization process of azo materials.

Azo materials are a class of materials with a molecular structure of $R_{1}-N=N-R_{2}$, which has two isomer structures of cis and trans, as shown in Figure 4; their photochromism is caused by the cis-trans isomerization of molecules under the action of light. The rearrangement of the molecules makes the material anisotropic, and the ordered and disordered arrangement of molecules forms a grating. When different polarized states of light are applied to the material, the molecules will reorient to realize the writing and erasing of information. The number of cis and trans isomers can be controlled by irradiating different wavelengths of light because the cis and trans isomers of azo materials have different absorption spectra. Because of the photothermal instability of material molecules, the recording performance of azo materials is easily restricted and affected by the environment.

In 1972, Paik et al. discovered that when compounds containing azo-aromatic groups were irradiated with light of appropriate wavelengths, trans-cis and cis-trans isomerization occurred through photochemical processes [20]. This discovery underscored the great potential of azo materials as a new optoelectronic material in optical information storage, all-optical modulation, spatial light modulator, and integrated optics.

According to the study of photoanisotropy, research on polarization holographic recording of azopolymer materials has been continuously developed. In 1984, Todorov et al. proposed a new type of polarization-sensitive holographic recording material of polyvinyl alcohol polymer doped with azo-dye methyl-orange and proved that the material based on photoinduced anisotropy was an efficient material for polarization holographic recording, allowing for multiple erasure and repeated recording [21]. The team then verified the properties of the polarization grating in the described medium. In 1987, M. Eich et al. proved that azo liquid crystal polymer had good performance as an optical holographic recording medium and optical element [22]. It could induce trans-cis isomerization of the liquid crystal contained azobenzene group at side-chains to make it appear in an anisotropic state and record this state at a certain temperature. When the temperature exceeded a specific value, the change in optical properties can be completely eliminated within a few seconds, showing its good erasing characteristics. In 1990, M. Eich further used polarized ultraviolet-visible spectroscopy to perform detailed experimental characterization of the optical holographic recording performance of azo liquid crystal polymers [23]. Then, W. M. Gibbons et al. used the polarization state of interference light to control the molecular orientation of the liquid crystal surface and formed a polarization grating with high diffraction efficiency through precise control of the spatial distribution of the liquid crystal molecular orientation [24].

In 1999, Birabassov et al. prepared a cellulose acetate film doped with azo dye Disperse Red 1 and used this material to provide a hologram with similar efficiency for the grating recorded with intensity and polarization modulation, storage, and reconstruction of the polarization state of elliptically polarized object light [25]. In 2002, Nikolova et al. explored the reflective polarization holographic properties of azo-side-chain polyester and recorded the polarization holographic gratings in thin samples using two circularly polarized waves [26]. In 2003, Ramanujam employed an evanescent field to realize high-resolution polarization holographic recording in an azobenzene polyester film material placed on the hypotenuse of a highly refractive prism and produced a polarization grating with a spatial frequency greater than 7000 lines/mm [27]. The two-photon effect of azobenzene polymer can increase the diffraction efficiency in the dark after the writing beams have been switched off.

Having a large refractive index modulation is important for polarization holographic recording of materials, and an elegant approach is to add photoreactive liquid crystals and azo-dyes as composites. Liquid crystals have demonstrated that they are sensitive to the polarization state of the recording waves. They can effectively enhance the diffraction efficiency under appropriate control of collective reorientation of the mesogenic terminal ends [28]. In 2005, Hiroshi et al. investigated the polarization holographic recording with two waves that had orthogonal linear polarizations in two samples of azobenzene-containing amorphous polymers and polymer dissolved liquid crystalline composites and used theoretical calculations to explain the diffraction properties of the two samples [29]. Meanwhile, they found that small input powers are sufficient for inducing a large refractive index modulation of approximately $5.0\times 10^{-3}$. In 2008, Pan Xu et al. described a liquid crystalline azobenzene polymer film image storage technology based on circular polarization holographic recording [30]. Two orthogonal circularly polarized 532 nm beams were used to store the image in a pure polarization hologram, and a 633 nm beam was adopted to reconstruct the stored image. A grating with high diffraction efficiency and long-term storage was obtained in a stable hologram. In 2010, A. Shishido reviewed the rewritable holographic recording material of liquid crystal polymer containing an azo side-chain, which has the recording ability of the polarization hologram [31]. In 2012, Arora et al. reported a polarization holographic grating recording in a liquid crystalline azo dye copolymer with a hidden helical superstructure [32]. The polymer effectively recorded the amplitude and polarization holographic gratings. Its polarization modulation results in reorientation of liquid crystal terminal ends, which can lead to a spatial modulation of the birefringence. The maximum achieved diffraction efficiency of first-order diffracted light was about 30%. Liquid crystalline azo dye is a good combination of the trans–cis isomerization and photo-orientation, used for polarization recording. In 2016, Martinez-Ponce et al. presented a theoretical and experimental polarimetric analysis on the three configurations traditionally used to form polarization gratings in a film made of an azobenzene-containing polymer within the frame of the Muller–Stokes formalism [33]. This work furthered understanding of the recording process in these types of photoanisotropic materials. A new type of self-organized material based on cholesteric networks filled with photoactive side-chain copolymer has been developed. Photochromic azobenzene-containing nematic copolymer is embedded in a cholesteric scaffold of a supramolecular helical structure of a cholesteric polymer network and utilized as a polarization-sensitive media for optical patterning. The high light-induced optical anisotropy comes from effective photo-orientation of side-chain fragments of the azobenzene-containing liquid crystalline polymer.

To enhance the photoinduced change in the refractive index, the fabrication of highly birefringent azo dye doped and azo liquid crystal control is a key process that can enhance the photoanisotropic of the material to improve the diffraction efficiency of polarization recording by the trans–cis isomerization and photo-orientation. Several approaches to obtain high-performance parameters of the Bragg holograms have been introduced [34]. Research on azo-based polymer materials continues to advance, and many new azo-containing composite materials, such as azo materials doped with nano-metal particles and organic-inorganic hybrid materials containing azo, have been widely researched. Although the polarization storage characteristics of these new materials require further study, they undoubtedly provide new ideas for the discovery of new azo optical storage materials.

### 3.2. Photopolymer Material

Photopolymer materials have attracted increasing attention because of the advantages such as low cost, easy preparation, flexible composition selection, and designability. Compared with other organic materials, photopolymers are widely used as a holographic recording material suitable for one-time writing and permanent reading. These materials not only have a high refractive index modulation and diffraction efficiency, but also can improve performance through doping, which gives them great advantages in terms of holographic storage.

**Figure 5:**
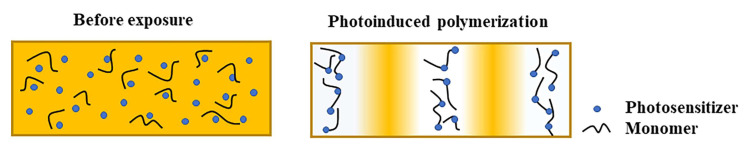
The process of photopolymerization for the formation of holographic recording gratings.

A typical photopolymer system generally includes the photosensitizer, monomer, and initiator. The photosensitizer and initiator can form a photosensitive initiation system because both can promote the polymerization of monomers during the photopolymerization process [35]. Photosensitive initiation systems are the dominant component of the initial process of the photochemical reaction. After photopolymerization, monomers polymerize to form a polymer substrate, and the monomers react with photosensitizers to form photoproducts. Different photosensitive initiation systems can be selected to obtain different sensitivity wavelengths and photoinitiation sensitivity, which also determines the polarization sensitivity of the material. The basic process of photopolymerization for the formation of holographic recording grating is shown in Figure 5. The monomer and photosensitizer are uniformly distributed in the material system before exposure. When light shines on the sample, the photosensitizer absorbs photons and is excited and then reacts with the monomer to generate light products. Under exposure, the monomers are consumed the fastest in the polymerization interference areas. The increasing consumption of monomers in the bright regions forms a concentration gradient that leads to the diffusion of monomers from the dark areas to the light ones. This ends when most of the monomers consumed in the material have been polymerized into chains. The material-dependent refractive indexes are different from each other, and a phase grating can be implemented to record optical information.

One type of polarization-sensitive holographic recording photopolymer material that has been studied extensively is polymethyl methacrylate organic polymer doped with phenanthrenequinone (PQ/PMMA). PQ/PMMA polymer was first proposed as a traditional holographic storage material and attracted attention. The main principle of recording holographic gratings is to use the polymerization reaction of the monomer and base material and the diffusion of the monomer material.

**Figure 6:**
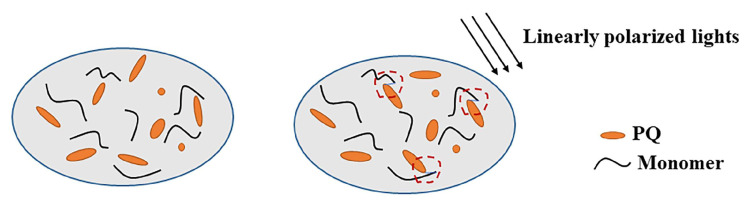
Photoreaction principle of phenanthrenequinone (PQ)/PMMA polymer.

The molecular structure of phenanthrenequinone has carbon-oxygen double bonds and carbon-carbon double bonds, which are highly conjugated two-dimensional planar structures. PQ molecules in different directions are randomly distributed in the material without illumination, and the material is isotropic, as presented in Figure 6. The orientation of the PQ molecules parallel to the light polarization state is more likely to react with the monomer in a specific area when irradiated with linearly polarized light. PQ absorbs photons and is excited to form a singlet excited state ${}^{1}{\mathrm{PQ}}^{*}$ under illumination. The singlet excited state changes to a triplet excited state ${}^{3}{\mathrm{PQ}}^{*}$, and hydrogen donors represented by the macromolecules RHare removed to generate active free radicals HPQ·. Among them, R represents the preformed PMMA polymer chain in the matrix of the layer. The free radical initiates the photo-linking reaction, which produces the photoproduct by a one-to-one linking reaction with the unsaturated vinyl c=c on the MMAmolecule [36,37]. This light reaction will cause polarization distribution, make the material anisotropic, and form a polarization grating to realize polarization holographic recording.

**Figure 7:**
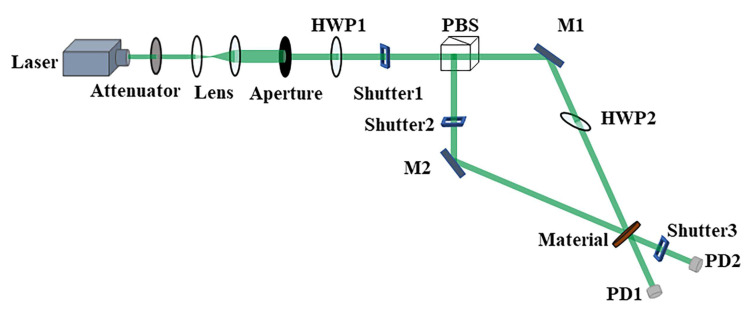
Experimental device for the diffraction efficiency of photopolymer materials. M, HWP, PBS, and PD are the mirror, half-wave plate, polarization beam splitter, and photodetector, respectively.

The polarization holographic recording performance of PQ/PMMA materials can be evaluated by parameters such as diffraction efficiency and photoinduced birefringence. The maximum diffraction efficiency of the material is an important criterion for the potential of the material to become a polarization holographic storage material. Diffraction efficiency is the ratio of the diffracted luminous flux of the polarized holographic image to the luminous flux incident on the material and is defined as follows:

(1) $$\eta =\frac{I_{1}}{I_{0}}$$

where $I_{1}$ is diffracted light intensity and $I_{0}$ is incident light intensity. The specific experimental device is shown in Figure 7.

Photoinduced birefringence is the macroscopic manifestation of the microscopic molecular anisotropy of the medium and is an important photophysical manifestation of anisotropic materials. It is mainly caused by the structural rearrangements of the photoproduct induced by the photochemical reaction of photopolarization-sensitive molecules [38]. When the material is irradiated with polarized light along the x-axis direction, its refractive index along the x-axis and y-axis directions is different because of the anisotropy of the material. This causes the material to produce birefringence. Photoinduced birefringence Δn is one of the key parameters for polarization-sensitive holographic recording material. For the PQ/PMMA material system, the structural rearrangement induced by the photochemical reaction of PQ molecules and MMA molecules causes photoinduced birefringence, which makes the material anisotropic.

When material absorption is ignored, the photoinduced birefringence (Δn) is usually given by [39]:

(2) $$\Delta n=n_{1}-n_{2}=\frac{\lambda }{\pi d}arcsin\sqrt{\frac{I_{T}}{I_{0}sin^{2}2\theta_{0}}}$$

where $n_{1}$, $n_{2}$ are the refractive index along and perpendicular to the polarization direction of the pump light, d is the thickness of the medium, λ is the wavelength of the pump laser, $I_{0}$, $I_{T}$ are the intensity of the probe wave before and after pumping exposure, and $\theta_{0}$ is the angle between the polarization direction of the pump laser and that of the probe laser, respectively.

In the early days, the research on the improvement of the holographic recording performance of photopolymer materials mainly focused on traditional holography. In 1969, Close et al. drew attention to photopolymers as holographic recording materials [40]. The material included a monomer mixture solution composed of acrylamide, barium acrylate, and lead acrylate and a photocatalyst solution that consisted of components such as methylene blue. Close et al. successfully recorded holograms using this material and described some characteristics. In 1975, Sadlej et al. added a polyvinyl alcohol adhesive on the basis of the original material system proposed by Close et al. [41]. This improvement made it possible to produce a stable dry photopolymer layer that improved diffraction efficiency and enhanced stability. Since then, researchers have continued to explore the photopolymer materials reported by Close et al. Photopolymer materials have been used widely in the field of holography, but the volume of polymer materials will shrink after storage. This volume shrinkage may lead to the deviation or distortion of the recorded information, which affects information readout. Moreover, a thicker sample leads to more obvious shrinkage effects. This property also seriously affects the storage capacity of photopolymer materials and hinders development in the holographic storage field.

Many groups are developing thicker photopolymers that inhibit volume shrinkage. After a long-term study by the Tomita research group in Japan, Naoaki et al. demonstrated a green permanent holographic storage with high diffraction efficiency and recording sensitivity in a methacrylate photopolymer film dispersed with titanium dioxide nanoparticles in 2002 [42]. The diffraction efficiency and recording sensitivity of the material increased significantly with increasing nanoparticle concentration. Volume shrinkage was suppressed during holographic exposure. This discovery provided a breakthrough for improving the volume shrinkage of photopolymer materials.

Since then, the research group has continued to work in related directions. In 2003, Tomita et al. proved a more than one-order-of-magnitude improvement of holographic recording sensitivities in the green channel by doping pyrromethene dye into silica nanoparticles dispersed in methacrylate photopolymer film [43]. In 2005, Tomita et al. proposed a new organic-inorganic nanocomposite photopolymer in which inorganic titanium dioxide or silicon dioxide nanoparticles were dispersed in methacrylate monomer syrup [44]. This photopolymer had permanent holographic storage capacity. The net diffraction efficiency was close to 100%, and the polymerization shrinkage was visibly suppressed. The following year, Tomita et al. used an electron probe microanalyzer to analyze the two-dimensional morphology and nanoparticle distribution formed in the hologram [45]. They used S atoms and Si atoms as label elements to identify the types of formed polymers and nanoparticles. The periodic densities of the formed polymers and nanoparticles were inconsistent. The results proved the mutual diffusion of monomer molecules and silica nanoparticles in the film under holographic two-beam interference exposure and further explained the mechanism of grating formation in nano-particle-doped photopolymers. The research of Tomita’s group improved the volume shrinkage characteristics of photopolymers and established the foundation for a wide application of photopolymers in volume holographic storage.

Phenanthraquinone-doped polymethyl methacrylate (PQ/PMMA) polymer materials have excellent properties. For example, the polymerization reaction of the monomer during the preparation process and the photochemical reaction during the exposure process can be separated, and thus, the shrinkage during the exposure process is negligible. The dual-polymer mode is adopted, and one of the polymers is employed to form the substrate of the material, which determines the thermal expansion coefficient and optical characteristics of the material. Another polymer is evenly distributed in the substrate and completes photopolymerization under light to modulate the refractive index. Therefore, some properties of the material are determined by just one of the polymers, which means that the specified parameters of the material can be adjusted without affecting the other properties. This increases the selection range of the material. These properties can be improved by doping other components and has received extensive attention especially in polarization holographic storage materials.

In 2009, Trofimova et al. prepared the PQ/PMMA material and applied it to polarization holographic recording [38]. Through infrared spectroscopy and other techniques, it was confirmed that the photochemical reaction product of PQ and the substrate caused the birefringence. The birefringence induced in the materials by linearly polarized radiation in the visible range reaches $1.4\times 10^{-4}$ (at room temperature) and $2.2\times 10^{-4}$ (≈50 °C) for 3–4 mol% phenanthrenequinone. Meanwhile, the relationship between the birefringence and the temperature of the PQ dye was studied along with the influence of external radiation on the birefringence. This was the first report on the photoinduced anisotropy of PQ/PMMA materials. In 2014, Lin et al. studied the characteristics of thick PQ/PMMA photopolymer materials for recording polarization holograms through experiments [46]. The hologram recordings of 2-mm thick PQ/PMMA samples were performed for experimental characterization with orthogonal linearly and circularly polarized waves. The thermal polymerization preparation method, however, made the content of photosensitizer PQ molecules in the PQ/PMMA material system low at room temperature. This resulted in low photosensitivity and refractive index modulation of the material, which did not meet the requirements of polarization holographic storage technology. Therefore, researchers have tried to improve the polarization holographic performance of PQ/PMMA materials in a variety of ways.

In the same year, C. Li et al. improved the photoinduced birefringence of the material by doping gold nanorods in PQ/PMMA and theoretically gave an explanation model of the working mechanism with gold nanorods [47]. The diffusion-promoting effect of gold nanorods increased the linearly polarized photoinduced birefringence by 38%. It was a good reference value for the development of the PQ/PMMA photopolymer system. Subsequently, Y. Liu et al. also proved that doping nanoparticles can improve the photoinduced birefringence of the materials by doping aluminum and silica nanoparticles [48,49]. The method of doping nano-components also has a good reference value for improving the recording performance of PQ/PMMA.

The structure and concentration of photosensitive molecules also has a great influence on the properties of polarization sensitive materials. In view of the limitation of the PQ concentration in PQ/PMMA, in 2011, T. Fukuda et al. developed a new photoinitiator AK1with a photoinduced birefringence effect [50]. The holographic recording properties of polarization-sensitive materials with PQ as the photosensitizer and AK1 as the photosensitizer were compared. The photoinduced birefringence of the polymer material with AK1 is more than four times that of the polarization-sensitive photopolymer material with PQ as the photosensitizer. In addition, the solution concentration of AK1 can be more than 20 times higher than that of PQ dissolved in the MMA solution. This new type of polarization-sensitive material with AK1 has higher photoinduced birefringence than that with PQ polarization-sensitive materials as photoinitiators are expected to improve the properties of polarized holographic recording materials. In 2013, they developed an improved polarization-sensitive photopolymer material with AK1-H [51]. With optimized photosensitizer AK1-H doped, the conventional material AK1 for vector wave memories (VWMs) was modified to improve signal stability and recording characteristics.

In 2017, Liu et al. used Irgacure 784 instead of PQ to investigate the holographic properties of Irgacure 784-doped polymethyl methacrylate photopolymer (Irgacure 784/PMMA) and compared it with PQ/PMMA [52]. The material had better performance in diffraction efficiency, refractive index modulation, and recording sensitivity. In addition, this material was sensitive to polarization. The polarization holographic recording was successfully performed with a 532 nm laser beam in Irgacure 784/PMMA. The team also studied the photo-initiator concentration of the Irgacure 784/PMMA photopolymer material and the influence of different intensities of light used in the recording process on the diffraction efficiency. This laid the foundation for further development of this material in polarization holographic recording.

In 2018, Fan et al. introduced benzyl methacrylate (BzMA) into PQ/PMMA photopolymers [53]. Using BzMA as a monomer, free radical copolymerization with MMA was performed to synthesize high-concentration PQ-doped poly(MMA-co-BzMA). After adding BzMA, the concentration of PQ in the polymer increased from 0.7 wt % to 1.15 wt %. Experiments showed that the photoinduced birefringence increased from $6.3\times 10^{-5}$ to $9.5\times 10^{-5}$, and the diffraction intensity of the photopolymer was significantly improved compared with PQ/PMMA, which demonstrated that it is an excellent polarization holographic storage material. In the same year, Fan et al. increased the concentration of PQ from 0.7 wt % to 1.3 wt % by introducing the THFMAcomonomer into the PQ/PMMA photoinduced polymer [54]. The diffraction intensity and photoinduced birefringence of the material were improved compared with those of the previously prepared materials.

Work to improve PQ/PMMA photopolymer materials has continued in recent years. In addition to these methods of changing the photosensitizer composition and doping other components, some researchers have made progress by improving the preparation process of the materials to increase the concentration of PQ molecules [55]. For photopolymer materials, to improve the polarization sensitivity of materials, it is useful to synthesize or search for more effective polarization sensitive photosensitizer molecules, optimize the polymer substrate with compatible co-polymer substrate, and dope helpful nano-components. More and more experiments have verified that this photopolymer material has a promising future in polarization holographic storage.

### 3.3. Photorefractive Polymer Material

The photorefractive effect is a nonlinear optical phenomenon in which the variation of the refractive index of photoelectric materials is caused by the spatial distribution of light intensity under light irradiation. In 1966, Ashkin et al. found that when they focused the laser beam on LiNbO_3_ and LiTaO_3_, the optical inhomogeneity of the crystal refractive index was observed and could be maintained for a long time without exposing the light [56]. In 1968, Chen et al. applied this phenomenon to holographic storage using single crystal lithium niobate as a holographic storage material and found that the formed hologram had high diffraction efficiency and thermal erasability [57]. At the same time, the physical mechanism of light-induced refractive index inhomogeneity was studied, and a drift model of light-excited carriers was proposed. This discovery laid the foundation for subsequent research on photorefractive materials in holographic storage.

During photorefraction, free carrier migration has three main mechanisms: diffusion, drift, and the photovoltaic effect [58]. Ferroelectric materials will produce an abnormal photovoltaic effect of photovoltaic current along the direction of spontaneous polarization under uniform illumination. Under the action of this special effect, a photovoltaic electric field (i.e., a space charge field) is formed in the photorefractive crystal. The spatially modulated space charge field is formed when the spatially oscillating circular photovoltaic current forms a spatially modulated phase grating in the crystal through the linear electro-optic effect. This produces an orthogonal polarization holographic recording.

The principle of photorefractive polymer materials is similar to that of inorganic crystals. Compared with inorganic crystals, photorefractive polymer materials have excellent nonlinear optical properties and electro-optical effects and are easy to prepare, which has attracted wide attention.

In 1991, Ducharme et al. observed the photorefractive effect in amorphous electro-optical materials [59]. The formed grating presented the characteristics of dynamic writing and erasing, strong electric field dependence, polarization anisotropy, and high diffraction efficiency. This was the first report on photorefractive polymer materials. Since then, researchers have conducted a series of studies and developed many new photorefractive polymer materials. In 2003, Kim et al. proposed a photorefractive polymer composite material containing a capture layer [60]. By introducing the capture layer, the recording stability, gain coefficient, and diffraction efficiency could be improved without sacrificing the growth rate of the grating. This method provided a new idea for the preparation of photorefractive polymer materials with high recording stability and high growth rate. In 2009, Salvador et al. developed a highly sensitive photorefractive polymer composite material and integrated it into a holographic optical coherent imaging system as a recording medium for holographic optical imaging [61]. The optical biopsy was successfully realized through this system. In 2012, Tsutsumi et al. proposed a method to quickly update the holographic image of a photorefractive polymer composite material based on poly(N-vinyl carbazole) [62]. The hologram of the object coin was clearly recorded in the photorefractive polymer composite material. The holographic image was reconstructed with the probe beam. In 2017, Ni et al. doped 2–8 wt% ZnS nanoparticles into a holographic polymer-dispersed liquid crystal (HPDLC) with a controlled distribution, which significantly reduced the driving voltage of HPDLCnanocomposites while maintaining high diffraction efficiency [63]. The composite demonstrated great potential in the holographic storage field.

At present, there are few reports on the application of photorefractive polymer materials in polarization holographic recording, but researchers have conducted many theoretical and experimental studies on the polarization properties of photorefractive polymer materials and have obtained some performance.

## 4. Conclusions

We report on the basic optical design of polarization holographic recording, as well as corresponding system demonstrations. Polarization holographic recording offers the best prospect for holographic data storage, including the fabrication of artificial anisotropic elements and polarization control. This includes the physical principles and high performance of polarization-sensitive media.

We present an overview of reported work on the polarization-sensitive materials and discuss three types of typical polarization-sensitive material in detail. We examine the key contributions of each work, and many of these suggestions have improved the different polarization-sensitive materials discussed in this work. Clearly, many materials have provided promising polarization-sensitive capacities and are being developed for polarization holographic application. We emphasize photopolymers in this review, as they offer many advantages and are widely used as holographic data storage media. In particular, we discuss the polarization-sensitive mechanism of the PQ/PMMA material, which offers an in-depth understanding of comprehensive improvements in different photopolymers.

Various approaches are summarized to improve performance in the field of polarization-sensitive holographic recording materials. Such improvements include (i) the introduction of the liquid crystal structure that betters the manipulated material polarization-sensitive properties; (ii) the introduction of nanoparticles that increase the absorption and promote effective photoconversion of photosensitive molecules; (iii) optimization of the photosensitizer composition structure and polymer composite substrate that improves the photoinduced birefringence of polarization-sensitive materials. The development of polarization holographic recording in optical holographic storage, optical element production, and other related fields needs continuous improvements.
